# *N*,*N*,*N*′,*N*′-Tetra­methyl­ethylene­diammonium tetra­chloridocobaltate(II)

**DOI:** 10.1107/S1600536810048749

**Published:** 2010-12-04

**Authors:** Russell G. Baughman, Rebecca S. Shane, James M. McCormick

**Affiliations:** a Department of Chemistry, Truman State University, Kirksville, MO 63501-4221, USA; b Department of Physics, Washington University, St Louis, MO 63130, USA

## Abstract

The asymmetric unit of the title compound, [(CH_3_)_2_NH(CH_2_)_2_NH(CH_3_)_2_][CoCl_4_], contains a tetra­chlorido­cobalt­ate(II) dianion and two halves of two centrosymmetric, crystallographically-independent, dications. One independent dication is disordered between two conformations in a 0.784 (13):0.216 (13) ratio. In the crystal, inter­molecular N—H⋯Cl hydrogen bonds link cations and anions into chains propagated in [0
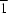
1]. These hydrogen bonds contribute to the distorted tetra­hedral geometry at the Co^II^ atom.

## Related literature

The synthesis of the title compound was modified from that of Szafran *et al.* (1998 ▶). Related tetra­methyl­ethylene­diammo­nium salts are listed in the Cambridge Structural Database (Allen, 2002 ▶).
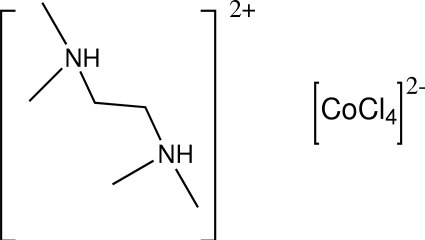


## Experimental

### 

#### Crystal data

(C_6_H_18_N_2_)[CoCl_4_]
*M* *_r_* = 318.95
Triclinic, 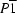

*a* = 6.9179 (3) Å
*b* = 8.2866 (3) Å
*c* = 13.4395 (5) Å
α = 72.188 (3)°
β = 87.292 (3)°
γ = 69.045 (3)°
*V* = 683.31 (5) Å^3^
*Z* = 2
Mo *K*α radiation
μ = 2.00 mm^−1^
*T* = 295 K
0.55 × 0.44 × 0.38 mm

#### Data collection

Bruker P4 diffractometer
Absorption correction: integration (*XSHELL*; Bruker, 1999 ▶) *T*_min_ = 0.378, *T*_max_ = 0.553
3003 measured reflections
2361 independent reflections
2207 reflections with *I* > 2σ(*I*)
*R*_int_ = 0.055
3 standard reflections every 100 reflections  intensity decay: 3.8%

#### Refinement

*R*[*F* ^2^ > 2σ(*F* ^2^)] = 0.035
*wR*(*F* ^2^) = 0.097
*S* = 1.08
2361 reflections
129 parameters
H-atom parameters constrained
Δρ_max_ = 0.53 e Å^−3^
Δρ_min_ = −0.50 e Å^−3^

### 

Data collection: *XSCANS* (Bruker, 1996 ▶); cell refinement: *XSCANS*; data reduction: *XSCANS*; program(s) used to solve structure: *SHELXS86* (Sheldrick, 2008 ▶); program(s) used to refine structure: *SHELXL97* (Sheldrick, 2008 ▶); molecular graphics: *SHELXTL/PC* (Sheldrick, 2008 ▶); software used to prepare material for publication: *SHELXTL/PC* and *SHELXL97*.

## Supplementary Material

Crystal structure: contains datablocks I, global. DOI: 10.1107/S1600536810048749/cv2786sup1.cif

Structure factors: contains datablocks I. DOI: 10.1107/S1600536810048749/cv2786Isup2.hkl

Additional supplementary materials:  crystallographic information; 3D view; checkCIF report

## Figures and Tables

**Table:** 

Co1—Cl1	2.2500 (8)
Co1—Cl2	2.2980 (7)
Co1—Cl3	2.2686 (8)
Co1—Cl4	2.2615 (8)

**Table:** 

Cl1—Co1—Cl4	115.50 (4)
Cl1—Co1—Cl2	106.29 (4)
Cl1—Co1—Cl3	106.99 (4)
Cl2—Co1—Cl3	107.06 (3)
Cl2—Co1—Cl4	112.81 (3)
Cl3—Co1—Cl4	107.76 (3)

**Table 2:** Hydrogen-bond geometry (Å, °)

*D*—H⋯*A*	*D*—H	H⋯*A*	*D*⋯*A*	*D*—H⋯*A*
N1*A*—H1*AD*⋯Cl2	0.91	2.31	3.170 (2)	157
N1*B*—H1*BD*⋯Cl3	0.91	2.37	3.222 (3)	155

**Fig. 1.**
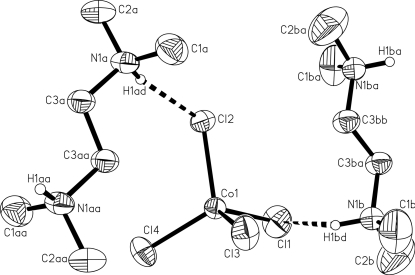
View of the title compound (asymmetric unit plus inversion-related pairs of both TMEDs) showing the atomic labeling [symmetry codes: (a; left TMED) 1-x, 1-y, -z; (b; right TMED) 1-x, -y, 1-z]. Only the major conformation of the disordered TMED cation is shown. Displacement ellipsoids are shown at 50% probability level. Amine H atoms involved in significant hydrogen bonding (dashed lines) are drawn as small spheres of arbitrary radius.

## supplementary crystallographic information

### Comment

During the evaluation of the generality of the procedure of Szafran, Pike, and Singh (1998) for Truman State University's inorganic chemistry course, the title compound, *N*,*N*,*N*',*N*'-tetramethylethylenediammonium (TMED) tetrachlorocobaltate(II), (I), was the unexpected product. A cobalt(III)-TMED complex had been anticipated. A search of the Cambridge Structural Database (v. 5.31; Allen, 2002) for TMED and TMED-related salts yielded 82 results (from monoatomic to complex anions); the structure of the [CoCl_4_]^2-^ salt has not been reported, and thus was deemed appropriate for determination.

Two different halves ("A" & "B" suffixes) of the cation are present in the asymmetric unit. The N/C/C/N sections of each cation are planar causing each half to be related to its partner half *via* a center of inversion in the middle of the cation. Evidence for different conformations of the "A" *versus* "B" TMED cations include the different methyl C distances from the respective N/C/C/N least-squares plane. The more distant methyl C atoms (C1A and C1B) are 1.313 (6) Å and 1.191 (4) Å, respectively, from their planes. Similarly, C2A and C2B are -0.419 (7) Å and -0.25 (1) Å, respectively, from their planes. Additionally, magnitudes of the corresponding torsion angles involving the methyls are somewhat comparable, but not equal.

The N atoms in the TMEDs shown in Fig. 1 are not symmetrically disposed about the [CoCl_4_]^2-^. The Co1···N1A and Co1···N1AA (= N1A at 1 - *x*, 1 - *y*, -*z*) distances are comparable [4.720 (2) Å and 4.808 (2) Å, respectively], while the Co1···N1B and Co1···N1BA (= N1B at 1 - *x*, -*y*, 1 - *z*) are quite different [4.143 (2) Å and 5.083 (2) Å, respectively] not only from each other, but also from the "A" TMED Co1···N distances.

Examination of the bond lengths and angles reveals numerous significant (\geq 3σ) differences between the "A" and "B" TMED cations. The "B" TMED exhibits disorder [0.784 (13); 0.216 (13)]. In both TMED cations the *E* conformation (likely due to the preference of dipoles within a molecule to oppose each other) of the methyls, nitrogen, and the amine H atoms shown in Fig. 1 contributes greatly not only to the presence of a center of inversion, but also to the one-dimensional hydrogen bonding present along [0–11].

A highly distorted tetrahedral geometry is present around the Co (*cf*. the six different Cl—Co—Cl angle values and four distances in Table 1). The ranges of distance and angle values are, respectively, 0.048 Å (\sim 64σ) and 9.21° (~230σ). Two of the Cl's in the [CoCl_4_]^2-^ moiety are involved in hydrogen bonding with amine H's in either the asymmetric unit or symmetry-related amine H's (Table 2). In both "A" and "B" cations, short (~2.3 Å) H-bond distances are noted for each hydrogen and are shown in Fig. 1. The strong hydrogen bonds (H1AD and H1BD with Cl2 and Cl3, respectively) are concomitant with the long Co1—Cl2 and Co1—Cl3 bond lengths. These interactions are undoubtedly the underlying cause of the severely distorted geometry of the [CoCl_4_]^2-^ anion.

### Experimental

The title compound was synthesized using a method parallel to that of Szafran, Pike, and Singh (1998) for the *trans*-dichloro *bis*- ethylenediamine cobalt(III) chloride using CoCl_2_^.^H_2_O and TMED for this work.

### Refinement

Approximate positions of the amine H's (H1AD & H1BD) and most of the methyl and methylene H's were first obtained from a difference map, then placed into idealized positions (C—H 0.96-0.97 Å; N—H 0.91 Å), and refined as riding, with U_iso_(H) = 1.2-1.5 *U*_eq_ of the parent atom.

In the final stages of refinement five reflections with very small or negative *F*_o_'s were deemed to be in high disagreement with their *F*_c_'s and were eliminated from final refinement.

### Crystal data

**Table:**

(C_6_H_18_N_2_)[CoCl_4_]	*Z* = 2
*M**_r_* = 318.95	*F*(000) = 326
Triclinic, *P*1	*D*_x_ = 1.550 Mg m^−^^3^
Hall symbol: -P 1	Mo *K*α radiation, λ = 0.71073 Å
*a* = 6.9179 (3) Å	Cell parameters from 100 reflections
*b* = 8.2866 (3) Å	θ = 10.4–21.8°
*c* = 13.4395 (5) Å	µ = 2.00 mm^−^^1^
α = 72.188 (3)°	*T* = 295 K
β = 87.292 (3)°	Block cut from larger crystal, blue
γ = 69.045 (3)°	0.55 × 0.44 × 0.38 mm
*V* = 683.31 (5) Å^3^	

### Data collection

**Table:**

Bruker P4 diffractometer	2207 reflections with *I* > 2σ(*I*)
Radiation source: normal-focus sealed tube	*R*_int_ = 0.055
graphite	θ_max_ = 25.0°, θ_min_ = 2.7°
θ/2θ scans	*h* = −8→1
Absorption correction: integration (XSHELL; Bruker, 1999)	*k* = −9→9
*T*_min_ = 0.378, *T*_max_ = 0.553	*l* = −15→15
3003 measured reflections	3 standard reflections every 100 reflections
2361 independent reflections	intensity decay: 3.8%

### Refinement

**Table:**

Refinement on *F*^2^	Secondary atom site location: difference Fourier map
Least-squares matrix: full	Hydrogen site location: inferred from neighbouring sites
*R*[*F*^2^ > 2σ(*F*^2^)] = 0.035	H-atom parameters constrained
*wR*(*F*^2^) = 0.097	*w* = 1/[σ^2^(*F*_o_^2^) + (0.0551*P*)^2^ + 0.3622*P*] where *P* = (*F*_o_^2^ + 2*F*_c_^2^)/3
*S* = 1.08	(Δ/σ)_max_ < 0.001
2361 reflections	Δρ_max_ = 0.53 e Å^−^^3^
129 parameters	Δρ_min_ = −0.50 e Å^−^^3^
0 restraints	Extinction correction: *SHELXL97* (Sheldrick, 2008), *F*_c_^*^=k*F*_c_[1+0.001x*F*_c_^2^λ^3^/sin(2θ)]^-1/4^
Primary atom site location: structure-invariant direct methods	Extinction coefficient: 0.075 (5)

### Special details

**Table:**

Geometry. All s.u.'s (except the s.u. in the dihedral angle between two l.s. planes) are estimated using the full covariance matrix. The cell s.u.'s are taken into account individually in the estimation of s.u.'s in distances, angles and torsion angles; correlations between s.u.'s in cell parameters are only used when they are defined by crystal symmetry. An approximate (isotropic) treatment of cell s.u.'s is used for estimating s.u.'s involving l.s. planes.
Refinement. Refinement of *F*^2^ against ALL reflections. The weighted *R*-factor, *wR*, and goodness of fit, *S*, are based on *F*^2^; conventional *R*-factors, *R*, are based on *F*, with *F* set to zero for negative *F*^2^. The threshold expression of *F*^2^ > 2σ(*F*^2^) is used only for calculating *R*-factors(gt) *etc*. and is not relevant to the choice of reflections for refinement. *R*-factors based on *F*^2^ are statistically about twice as large as those based on *F*, and *R*-factors based on ALL data will be even larger.

### Fractional atomic coordinates and isotropic or equivalent isotropic displacement parameters (Å^2^)

**Table:**

	*x*	*y*	*z*	*U*_iso_*/*U*_eq_	Occ. (<1)
Co1	0.08521 (5)	0.39063 (4)	0.24591 (2)	0.03454 (18)	
Cl1	−0.07188 (15)	0.25750 (14)	0.37896 (7)	0.0711 (3)	
Cl2	0.28898 (11)	0.16529 (9)	0.17970 (5)	0.0440 (2)	
Cl3	0.30208 (13)	0.48322 (11)	0.31660 (6)	0.0556 (2)	
Cl4	−0.12874 (12)	0.63518 (11)	0.12131 (7)	0.0578 (3)	
N1A	0.6224 (3)	0.2465 (3)	0.02177 (19)	0.0385 (5)	
H1AD	0.5067	0.2282	0.0497	0.046*	
C1A	0.7839 (5)	0.1787 (5)	0.1090 (3)	0.0612 (9)	
H1AA	0.8191	0.0502	0.1406	0.092*	
H1AB	0.7315	0.2393	0.1606	0.092*	
H1AC	0.9053	0.2028	0.0823	0.092*	
C2A	0.6891 (5)	0.1436 (4)	−0.0545 (3)	0.0556 (8)	
H2AA	0.5776	0.1831	−0.1065	0.083*	
H2AB	0.7257	0.0161	−0.0187	0.083*	
H2AC	0.8072	0.1654	−0.0878	0.083*	
C3A	0.5641 (4)	0.4454 (4)	−0.0337 (2)	0.0415 (6)	
H3AA	0.4861	0.4776	−0.0994	0.050*	
H3AB	0.6888	0.4738	−0.0493	0.050*	
N1B	0.3531 (4)	0.2003 (3)	0.54857 (19)	0.0469 (6)	
H1BD	0.2985	0.2773	0.4837	0.056*	
C1B	0.5067 (8)	0.2606 (6)	0.5827 (3)	0.0840 (13)	
H1BA	0.4372	0.3765	0.5935	0.126*	
H1BB	0.6036	0.2716	0.5299	0.126*	
H1BC	0.5794	0.1735	0.6471	0.126*	
C2B	0.1808 (8)	0.2172 (9)	0.6196 (4)	0.0998 (16)	
H2BA	0.1246	0.3391	0.6233	0.150*	
H2BB	0.2324	0.1338	0.6883	0.150*	
H2BC	0.0741	0.1893	0.5932	0.150*	
C3BA	0.4254 (8)	0.0107 (5)	0.5425 (3)	0.0415 (15)	0.784 (13)
H3BA	0.3081	−0.0170	0.5282	0.050*	0.784 (13)
H3BB	0.4934	−0.0736	0.6085	0.050*	0.784 (13)
C3BB	0.542 (2)	0.0458 (19)	0.5298 (11)	0.040 (5)	0.216 (13)
H3BC	0.6148	−0.0385	0.5948	0.048*	0.216 (13)
H3BE	0.6355	0.0950	0.4876	0.048*	0.216 (13)

### Atomic displacement parameters (Å^2^)

**Table:**

	*U*^11^	*U*^22^	*U*^33^	*U*^12^	*U*^13^	*U*^23^
Co1	0.0331 (2)	0.0344 (3)	0.0343 (2)	−0.01054 (16)	0.00261 (14)	−0.01021 (16)
Cl1	0.0643 (5)	0.0794 (6)	0.0662 (5)	−0.0350 (5)	0.0280 (4)	−0.0102 (5)
Cl2	0.0475 (4)	0.0414 (4)	0.0432 (4)	−0.0128 (3)	0.0093 (3)	−0.0182 (3)
Cl3	0.0701 (5)	0.0565 (5)	0.0496 (4)	−0.0363 (4)	−0.0092 (4)	−0.0113 (3)
Cl4	0.0473 (4)	0.0481 (4)	0.0596 (5)	−0.0079 (3)	−0.0117 (3)	−0.0002 (4)
N1A	0.0317 (10)	0.0304 (11)	0.0541 (13)	−0.0110 (9)	0.0104 (9)	−0.0154 (10)
C1A	0.0536 (19)	0.057 (2)	0.064 (2)	−0.0098 (15)	−0.0066 (16)	−0.0161 (16)
C2A	0.0516 (17)	0.0465 (17)	0.073 (2)	−0.0108 (14)	0.0096 (15)	−0.0338 (16)
C3A	0.0430 (14)	0.0333 (13)	0.0477 (15)	−0.0120 (11)	0.0111 (12)	−0.0149 (12)
N1B	0.0594 (15)	0.0358 (13)	0.0371 (12)	−0.0050 (11)	−0.0009 (10)	−0.0135 (10)
C1B	0.121 (4)	0.080 (3)	0.066 (2)	−0.061 (3)	−0.009 (2)	−0.012 (2)
C2B	0.088 (3)	0.145 (5)	0.083 (3)	−0.048 (3)	0.031 (3)	−0.055 (3)
C3BA	0.046 (3)	0.037 (2)	0.045 (2)	−0.0160 (17)	0.0098 (18)	−0.0166 (15)
C3BB	0.031 (8)	0.040 (7)	0.047 (8)	−0.009 (6)	0.012 (5)	−0.014 (6)

### Geometric parameters (Å, °)

**Table:**

Co1—Cl1	2.2500 (8)	N1B—C1B	1.469 (5)
Co1—Cl2	2.2980 (7)	N1B—C2B	1.485 (5)
Co1—Cl3	2.2686 (8)	N1B—C3BA	1.496 (4)
Co1—Cl4	2.2615 (8)	N1B—C3BB	1.542 (13)
N1A—C2A	1.483 (4)	N1B—H1BD	0.9100
N1A—C1A	1.487 (4)	C1B—H1BA	0.9600
N1A—C3A	1.496 (3)	C1B—H1BB	0.9598
N1A—H1AD	0.9100	C1B—H1BC	0.9599
C1A—H1AA	0.9600	C2B—H2BA	0.9600
C1A—H1AB	0.9600	C2B—H2BB	0.9600
C1A—H1AC	0.9601	C2B—H2BC	0.9600
C2A—H2AA	0.9601	C3BA—C3BA^ii^	1.510 (9)
C2A—H2AB	0.9600	C3BA—H3BA	0.9600
C2A—H2AC	0.9599	C3BA—H3BB	0.9601
C3A—C3A^i^	1.509 (5)	C3BB—C3BB^ii^	1.51 (3)
C3A—H3AA	0.9700	C3BB—H3BC	0.9600
C3A—H3AB	0.9700	C3BB—H3BE	0.9600
			
Cl1—Co1—Cl4	115.50 (4)	N1B—C2B—H2BA	109.6
Cl1—Co1—Cl2	106.29 (4)	N1B—C2B—H2BB	109.4
Cl1—Co1—Cl3	106.99 (4)	H2BA—C2B—H2BB	109.5
Cl2—Co1—Cl3	107.06 (3)	N1B—C2B—H2BC	109.4
Cl2—Co1—Cl4	112.81 (3)	H2BA—C2B—H2BC	109.5
Cl3—Co1—Cl4	107.76 (3)	H2BB—C2B—H2BC	109.5
C1A—N1A—C2A	111.2 (2)	C3BB—C3BA—C3BB^ii^	90.0 (15)
C1A—N1A—C3A	112.5 (2)	C3BB—C3BA—N1B	74.6 (8)
C2A—N1A—C3A	109.8 (2)	C3BB^ii^—C3BA—N1B	131.5 (8)
C2A—N1A—H1AD	107.6	C3BB—C3BA—C3BA^ii^	51.4 (9)
C1A—N1A—H1AD	107.7	N1B—C3BA—C3BA^ii^	110.7 (4)
C3A—N1A—H1AD	107.6	C3BB—C3BA—H3BA	158.9
N1A—C1A—H1AA	109.4	C3BB^ii^—C3BA—H3BA	71.7
N1A—C1A—H1AB	109.5	N1B—C3BA—H3BA	109.6
H1AA—C1A—H1AB	109.5	C3BA^ii^—C3BA—H3BA	109.6
N1A—C1A—H1AC	109.5	C3BB—C3BA—H3BB	88.9
H1AA—C1A—H1AC	109.5	C3BB^ii^—C3BA—H3BB	115.9
H1AB—C1A—H1AC	109.5	N1B—C3BA—H3BB	109.6
N1A—C2A—H2AA	109.4	C3BA^ii^—C3BA—H3BB	109.2
N1A—C2A—H2AB	109.6	H3BA—C3BA—H3BB	108.1
H2AA—C2A—H2AB	109.5	C3BB^ii^—C3BA—H3BC	107.1
N1A—C2A—H2AC	109.4	N1B—C3BA—H3BC	93.1
H2AA—C2A—H2AC	109.5	C3BA^ii^—C3BA—H3BC	77.4
H2AB—C2A—H2AC	109.5	H3BA—C3BA—H3BC	150.8
N1A—C3A—C3A^i^	110.3 (3)	C3BA—C3BB—C3BA^ii^	90.0 (15)
N1A—C3A—H3AA	109.7	C3BA—C3BB—C3BB^ii^	51.5 (12)
C3A^i^—C3A—H3AA	109.8	C3BA—C3BB—N1B	69.3 (8)
N1A—C3A—H3AB	109.5	C3BA^ii^—C3BB—N1B	130.1 (12)
C3A^i^—C3A—H3AB	109.4	C3BB^ii^—C3BB—N1B	106.5 (14)
H3AA—C3A—H3AB	108.1	C3BA—C3BB—H3BB	46.1
C1B—N1B—C2B	109.6 (3)	C3BA^ii^—C3BB—H3BB	108.4
C1B—N1B—C3BA	117.8 (3)	C3BB^ii^—C3BB—H3BB	79.3
C2B—N1B—C3BA	106.6 (4)	N1B—C3BB—H3BB	89.6
C1B—N1B—C3BB	85.6 (7)	C3BA—C3BB—H3BC	93.1
C2B—N1B—C3BB	137.0 (7)	C3BA^ii^—C3BB—H3BC	115.0
C1B—N1B—H1BD	107.5	C3BB^ii^—C3BB—H3BC	111.4
C2B—N1B—H1BD	107.3	N1B—C3BB—H3BC	111.2
C3BA—N1B—H1BD	107.5	H3BB—C3BB—H3BC	47.0
C3BB—N1B—H1BD	105.5	C3BA—C3BB—H3BE	155.7
N1B—C1B—H1BA	109.4	C3BA^ii^—C3BB—H3BE	71.7
N1B—C1B—H1BB	109.5	C3BB^ii^—C3BB—H3BE	108.8
H1BA—C1B—H1BB	109.5	N1B—C3BB—H3BE	110.1
N1B—C1B—H1BC	109.5	H3BB—C3BB—H3BE	154.5
H1BA—C1B—H1BC	109.5	H3BC—C3BB—H3BE	108.8
H1BB—C1B—H1BC	109.5		
			
N1A—C3A—C3A^i^—N1A^i^	180.0	C3BB—N1B—C3BA—C3BA^ii^	−37.3 (9)
C1A—N1A—C3A—C3A^i^	−73.0 (4)	C3BB^ii^—C3BA—C3BB—C3BA^ii^	0.001 (2)
C2A—N1A—C3A—C3A^i^	162.5 (3)	N1B—C3BA—C3BB—C3BA^ii^	−133.5 (9)
N1B—C3BA—C3BA^ii^—N1B^ii^	180.0	N1B—C3BA—C3BB—C3BB^ii^	−133.5 (9)
N1B—C3BB—C3BB^ii^—N1B^ii^	180.0	C3BA^ii^—C3BA—C3BB—C3BB^ii^	−0.001 (2)
C1B—N1B—C3BA—C3BA^ii^	−66.5 (5)	C3BB^ii^—C3BA—C3BB—N1B	133.5 (9)
C1B—N1B—C3BB—C3BB^ii^	−169.3 (14)	C3BA^ii^—C3BA—C3BB—N1B	133.5 (9)
C2B—N1B—C3BA—C3BA^ii^	170.0 (4)	C1B—N1B—C3BB—C3BA	154.4 (8)
C2B—N1B—C3BB—C3BB^ii^	76.5 (15)	C2B—N1B—C3BB—C3BA	40.2 (13)
C1B—N1B—C3BA—C3BB	−29.1 (9)	C1B—N1B—C3BB—C3BA^ii^	−134.0 (19)
C2B—N1B—C3BA—C3BB	−152.7 (9)	C2B—N1B—C3BB—C3BA^ii^	111.8 (16)
C1B—N1B—C3BA—C3BB^ii^	−105.0 (15)	C3BA—N1B—C3BB—C3BA^ii^	72 (2)
C2B—N1B—C3BA—C3BB^ii^	131.5 (15)	C3BA—N1B—C3BB—C3BB^ii^	36.3 (11)
C3BB—N1B—C3BA—C3BB^ii^	−75.8 (19)		

Symmetry codes: (i) −*x*+1, −*y*+1, −*z*; (ii) −*x*+1, −*y*, −*z*+1.

### Hydrogen-bond geometry (Å, °)

**Table:**

*D*—H···*A*	*D*—H	H···*A*	*D*···*A*	*D*—H···*A*
N1A—H1AD···Cl2	0.91	2.31	3.170 (2)	157
N1B—H1BD···Cl3	0.91	2.37	3.222 (3)	155
